# A Pharmacokinetic Study of the Interaction Between Regorafenib and Paracetamol in Male Rats

**DOI:** 10.3390/pharmaceutics16111387

**Published:** 2024-10-28

**Authors:** Agnieszka Karbownik, Danuta Szkutnik-Fiedler, Filip Otto, Anna Wolc, Tomasz Grabowski, Zuzanna Maciejewska, Aleksandra Borycka, Edyta Szałek

**Affiliations:** 1 Department of Clinical Pharmacy and Biopharmacy, Poznań University of Medical Sciences, Rokietnicka 3, 60-806 Poznań, Poland; dszkutnik@ump.edu.pl (D.S.-F.); f.otto@ump.edu.pl (F.O.); eszalek@ump.edu.pl (E.S.); 2 Department of Animal Science, Iowa State University, 239E Kildee Hall, Ames, IA 50011, USA; awolc@iastate.edu; 3 Hy-Line International, Research and Development, 2583 240th Street, Dallas Center, IA 50063, USA; 4 Department of Inorganic Chemistry, Faculty of Pharmacy, Medical University of Gdańsk, M. Skłodowskiej-Curie 3a, 80-210 Gdańsk, Poland; tomasz.grabowski@gumed.edu.pl; 5 Students Scientific Association at the Department of Clinical Pharmacy and Biopharmacy, Faculty of Pharmacy, Poznań University of Medical Sciences, Rokietnicka 3, 60-806 Poznań, Poland; zuzannamaciejewska@poczta.onet.pl (Z.M.); olaborycka16@interia.pl (A.B.)

**Keywords:** drug–drug interaction, pharmacokinetics, regorafenib, paracetamol

## Abstract

**Background**: In clinical practice, the prevalent problem of polypharmacy could result in increased risks of drug–drug interactions. Regorafenib (REG) is commonly co-administered with paracetamol (PA) as a treatment protocol in cancer patients with pain therapy. **Purpose**: This study aimed to demonstrate the effect of paracetamol on the pharmacokinetic parameters of regorafenib and its metabolites following a single administration of both substances in rats. Additionally, the influence of REG and its metabolites on the pharmacokinetics of paracetamol was also determined. **Methods**: Twenty-four rats were divided randomly into three groups: REG group (II_REG_, regorafenib 20 mg/kg, *n* = 8), PA group (III_PA_, paracetamol 100 mg/kg, *n* = 8), and REG+PA co-administration group (I_REG+PA_, REG 20 mg/kg and PA 100 mg/kg, *n* = 8). The concentrations of regorafenib, regorafenib-*N*-oxide (M-2), and *N*-desmethyl-regorafenib-*N*-oxide (M-5) were determined using ultra-performance liquid chromatography-tandem mass spectrometry (UPLC–MS/MS). The plasma concentrations of PA and its glucuronide (GPA) and sulfate (SPA) metabolites were measured using the validated high-performance liquid chromatography method with ultraviolet detection (HPLC–UV). The pharmacokinetic parameters were calculated using a non-compartmental model. The statistical evaluation was performed in the SAS program. **Results**: After the administration of PA, the C_max_ and AUC_0–∞_ of REG increased by 890% and 1140%, respectively; for M-2, they increased by 220% and 170%, and for M-5, by 2130% and 1730% (C_max_ and AUC_0–∞_, respectively). A difference in the ratio of M-2/REG for AUC_0–∞_ and C_max_ between the groups was observed, but not for M-5/REG. The AUC_0–∞_ for PA and GPA decreased by 20.7% and 51.1%, respectively, when PA was co-administered with REG. But the AUC_0–∞_ for SPA increased by 91.35% in the I_REG+PA_ group. A difference in the ratio of GPA/PA for C_max_ and for SPA/PA for AUC_0–t_ and AUC_0–∞_ between the groups was observed. **Conclusions**: Paracetamol increased the plasma exposure of regorafenib, M-2, and M-5, which may exacerbate the drug’s side effects. In contrast, REG reduced paracetamol exposure and contributed to its faster elimination, which may reduce the analgesic and antipyretic effects of paracetamol. These findings suggest clinical relevance for oncology patients requiring analgesic treatment.

## 1. Introduction

Tyrosine kinase inhibitors (TKIs) are first- or second-line drugs in anticancer therapy. They inhibit the growth, proliferation, differentiation, and metastasis of cancer cells. They are approved by the Food and Drug Administration for the treatment of a wide variety of cancers, including non-small-cell lung cancer, chronic myeloid leukemia, thyroid cancer, renal cell carcinoma, metastatic breast cancer, as well as hepatocellular carcinoma and metastatic colorectal cancer. Regorafenib is a multi-target inhibitor of various tyrosine kinases (EGFRi, PDGFRi, and FGFRi) used in the treatment protocols for the above-mentioned cancers [1]. Administered orally, this cutting-edge chemotherapeutic agent inhibits multiple signaling pathways responsible for angiogenesis and oncogenesis, which would otherwise provide an optimal microenvironment for the developing tumor [2]. In addition, it has also been approved for the treatment of advanced-stage hepatocellular carcinoma (HCC) in patients previously treated with sorafenib. Regorafenib has two active metabolites, M-2 (regorafenib *N*-oxide) and M-5 (regorafenib-*N*-desmethylated *N*-oxide), which inhibit the activity of RET, VEGFR 1-3, KIT, FGFR1, FGFR, and many other diphenylurea kinases, such as RAF-1, BRAF, and BRAFV600E, among others. It comes in oral form, where the standard dose is 160 mg once daily. Its average bioavailability after administration in tablet form is 69%. It is mainly metabolized by cytochrome CYP3A4 and UGT1A9 and excreted in the feces. To a significant extent, regorafenib, as well as its two active metabolites, bind to plasma proteins (99.5%). Based on population studies, no significant effect of gender, age, and body weight on the pharmacokinetic parameters was noted [3,4]. Preclinical in vivo and in vitro studies confirm the broad-spectrum anticancer activity of regorafenib. This is due to its inhibition of a wide variety of signaling pathways in cancer cells [5]. Clinical trials are currently underway for the use of regorafenib in the treatment of clear cell renal cell carcinoma, gastric cancer, biliary tract cancer, sarcomas, and gliomas [6]. It has been shown that regorafenib as well as its active metabolite M-2 inhibit the glucuronidation process mediated by UGT1A1 and UGT1A9, while M-5 inhibits only UGT1A1. Both metabolites are weak substrates of P-glycoprotein, while M-5 is a weak substrate of BCRP. Moreover, regorafenib is also a substrate and an inhibitor of P-glycoprotein and BCRP [4]. An increase in the severity of cancer is very often associated with the need to use analgesics to reduce the severity of cancer pain. Moreover, in about 40% of patients, pain is not properly controlled. Due to its high safety profile, paracetamol is the most commonly used analgesic and antipyretic. It is prescribed at each of the three levels of the analgesic ladder and shows hyper-additive synergism with opioid drugs. Despite many studies, its mechanism of action is still not fully understood. The most well-known action of paracetamol is the inhibition of cyclooxygenases (COX-1, COX-2, and COX-3). The drug affects serotonergic glia and the endocannabinoid system. It acts on L-arginine in the nitric oxide synthesis. It also interacts with the voltage-gated Kv7 potassium channels and transient receptor potential (TRP) channels and blocks the Cav 3.2-type T-type calcium channels [7]; it also inhibits the expression of P-glycoprotein in the gut. It is metabolized in the liver. It can undergo sulfuric acid coupling by SULT1A1, 1A3/4, and 1E1, glucuronidation by UGT1A1 and UGT1A6 [8], and oxidation by cytochrome P450 [9], mainly CYP2E1. CYP2E1 is responsible for forming toxic NAPQI (*N*-acetyl-*p*-benzoquinone imine), which can contribute to liver damage [8]. The metabolite of paracetamol resulting from sulfation also exhibits minimal substrate affinity for BCRP. Therefore, co-administration with a drug that inhibits its elimination from the body may pose a risk of accumulation of paracetamol sulfate in the system. However, for other metabolites, such as paracetamol glucuronide, NAPQI, or NAPQI-GSH adducts, such data are lacking [10]. It has been shown that tyrosine kinase inhibitors, such as erlotinib and lapatinib, block the glucuronidation of paracetamol and contribute to its increased blood levels [11,12]. Due to the effects of both regorafenib and paracetamol on P-glycoprotein, the metabolism of both drugs by cytochrome P450 as well as glucuronidation, and the high likelihood of a patient using the two drugs simultaneously, there is a high risk of drug–drug interactions.

**Figure 1:**
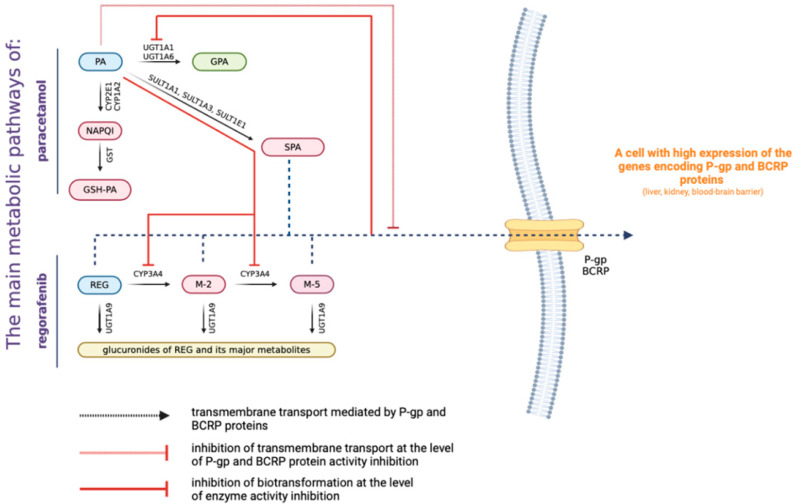
Pharmacokinetic interaction of paracetamol and regorafenib.

Based on the above arguments, the purpose of this study was to investigate the pharmacokinetic interaction of regorafenib–paracetamol after a single administration in healthy rats. The findings are expected to clarify the preclinical pharmacokinetic benefits or risks associated with using the regorafenib–paracetamol combination and provide a baseline for their concomitant clinical administration. The pharmacokinetic interaction of paracetamol with regorafenib is presented in Figure 1.

## 2. Materials and Methods

### 2.1. Chemicals and Reagents

REG (CAS No. 755037-03-7), M-2, M-5, REG-d3 (internal standard for the quantification of REG as well as M-2 and M-5), paracetamol glucuronide (GPA), and paracetamol sulfate (SPA) were purchased from LGC Standards (Łomianki, Poland). PA (CAS number 103-90-2), LC–MS grade methanol (MeOH), acetonitrile (ACN), water, formic acid (FA), glacial acetic acid, sodium sulfate, perchloric acid, theophylline (internal standard for the quantification of PA as well as GPA and SPA), sodium hydroxide, and dimethyl sulfoxide (DMSO) were purchased from Sigma-Aldrich (Saint Louis, MO, USA). The water used in the mobile phase was deionized, distilled, and filtered through a Millipore system (Direct Q3, Millipore, Burlington, MA, USA) before use. All chemicals and solvents were analytical grade.

REG (Stivarga^®^, batch No. BXJRJJ1) was purchased from Bayer Poland Sp. z o.o (Warsaw, Poland). PA (Pedicetamol^®^, batch No. K003) was purchased from Sequoia sp. z o.o. (Warsaw, Poland). Drug-free rat plasma was obtained from the Anima Sp. z o. o. SK & ViVARI s.c. (Warszawa, Poland).

### 2.2. Animals

Rats (*Rattus norvegicus*, outbred Wistar stock, males, 14 weeks old) were obtained from Anima Sp. z o.o. SK & ViVARI s.c. They were acclimated for one week in a temperature- and humidity-controlled environment with a 12 h light/dark cycle and had *ad libitum* access to food and water. The experimental protocol and procedures were approved by the Local Ethics Committee (Department of Animal Physiology and Biochemistry, Poznań University of Life Sciences, Wołyńska 35, 60-637 Poznań, Poland; approval no. 86/2022).

### 2.3. Animal Study Protocol

The rats were divided into three groups: one group received REG and PA (I_REG+PA_), another group received REG (II_REG_), and the last group received PA (III_PA_). REG was solubilized to form a clear solution for the dosing and was prepared in 10% DMSO solution (20 mg/kg body weight (b.w.)) [13,14]. Paracetamol was administered as an oral solution (Pedicetamol^®^, 100 mg/mL) at 100 mg/kg b.w. [15] to the I_REG+PA_ and III_PA_ groups. The drugs and vehicle were administered directly into the animals’ stomachs using a gastric probe. Animals received drugs in a sequential manner.

The blood samples (100 µL) were collected in heparinized tubes (containing 30 IU heparin sodium) via the tail vein at pre-dose, 0.25, 0.5, 1, 2, 3, 5, 7, 9, 24, 26, 48, and 72 h for REG and 0.083, 0.25, 0.50, 1, 1.5, 2, 4, 6, and 8 h for PA, and centrifuged at 2880× *g* for 10 min at 4 °C. Blood samples were stored at −80 °C until analysis.

In line with the decision of the Local Ethics Committee, pharmacokinetic data from the control group (II_REG_), which received REG alone, and from the III_PA_ group were adopted from a previous project examining pharmacokinetic interactions between REG and atorvastatin (ATO) [14] and between sorafenib and paracetamol [16]. This approach reduced the number of animals involved in the experiment, adhering to the principles of the 3R concept. However, the adoption of these results was limited due to the use of drugs (same manufacturer, same dose) from different batches. No other experimental conditions were altered.

### 2.4. Regorafenib and Paracetamol Analysis in Blood Samples

The analytical methods were validated according to the European Medicines Agency guidelines [17]. The statistical parameters for each compound are expressed as percentage ranges across four different concentrations: LLOQ (lower limit of quantification), 3xLLOQ, medium QC (medium concentration of quality control solution; concentration within 30–50% of the calibration curve), and high QC (high concentration of quality control solution; concentration at or above 75% of the calibration curve).

### 2.5. HPLC–UV Assay

The concentrations of PA, GPA, and SPA were assayed using high-performance liquid chromatography with ultraviolet detection (HPLC–UV) [18]. The column temperature was maintained at 25 °C, the analytical wavelength (λ_max_) was set at 254 nm, and the injection volume was 50 μL. Theophylline was used as the IS.

The total time of analysis for each run was 10 min. Separation was achieved by isocratic elution of the mobile phase, comprising acetonitrile (93:7, *v*/*v*) and sodium sulfate 0.05 M pH 2.2 (adjusted with 85% orthophosphoric acid) at a flow rate of 1.0 mL/min through a BDS Hypersil^®^ C18 column (150 mm × 4.6 mm, 5.0 μm particle size) (Thermo Electron Corporation^®^, Waltham, MA, USA).

The calibration curve for PA was linear within the range of 1.0–65.0 µg/mL (*r* = 0.999), for GPA within the range of 1.0–60.0 µg/mL (*r* = 0.998), and for SPA within the range of 1.0–50.0 µg/mL (*r* = 0.997). The high precision (coefficient of variation, CV < 11.0%) and accuracy (%bias ≤ 12.0%) of the applied methodology were confirmed for analytes. The lower limit of quantification (LLOQ) was 1.0 µg/mL for PA, GPA, and SPA. The limit of detection (LOD) was 0.1 µg/mL for PA, GPA, and SPA.

### 2.6. LC–MS/MS Assay

The quantification of REG, M-2, and M-5 was archived by an established LC–MS/MS system consisting of a Xevo TQ-S-micro triple quadrupole mass spectrometer coupled with a UPLC Acquity I-class PLUS (Waters Corporation, Milford, MA, USA) and the Waters Software MassLynx V4.2 SCN1017 Software. A Cortecs UPLC C18 column (2.1 × 50 mm, 1.6 μm) was equipped with an Acquity UPLC BEH C18 VanGuard pre-column (2.1 × 5 mm, 3/Pk, Waters Corporation). The injection volume was 5 μL. The temperature of the autosampler was 4 °C, and the temperature of the column was 40 °C. Mobile phase A comprised 0.1% aqueous FA solution (*v*/*v*) and phase B of ACN:MeOH and 0.1% FA aqueous solution at 1:3 (*v*/*v*) ratio. The total run time was 5 min.

The gradient was as follows: 0–0.5 min, with 10% B; 0.5–3 min, 95% B; 3–4 min, 95% B; 4–4.1 min, 10% B; and 4.1–5 min, 10% B. The flow rate was 0.4 mL/min. Positive electrospray ionization (ESI) multiple reaction monitoring (MRM) experiments were conducted for sample analysis. The Xevo TQ-S Micro Mass spectrometer (Waters) was run in the positive ion mode and configured in the multiple reaction monitoring mode for the detection of REG, M-2, M-5, and isotope-labeled REG-d3. The Xevo TQ-S micro mass spectrometer settings were used: source temperature 150 °C, desolvation temperature 600 °C, nitrogen gas flow 900 L/h, and capillary voltage 3.6 kV. Transition ion pairs (parent *m/z* → daughter *m/z*) were identified using the MRM mode for the following compounds: 482.95 → 270.08 and 288.02 for REG; 499.00 → 304.01, 252.16, and 229.00 for M-2; 485.94 → 202.02 and 228.98 for M-5; and 486.02 → 273.07 for IS.

The precision for REG, M-2, and M-5 was 4.8–17.2, 5.3–18.2, and 5.3–18.2%, respectively, while the accuracy for REG, M-2, and M-5 was −16.1–7.9, −18.8–10.4, and −18.8–10.4 %bias, respectively. The linear correlation coefficient (*r*) values of the regression equations were as follows: for REG, *r* = 0.998 (in the concentration range of 0.1–25 µg/mL); for M-2, *r* = 0.997 (in the concentration range of 5–2500 ng/mL); and for M-5, *r* = 0.997 (in the concentration range of 1–850 ng/mL). The lower limit of quantification (LLOQ) was 0.1 µg/mL, 5 ng/mL, and 1 ng/mL for REG, M-2, and M-5, respectively. The limit of detection (LOD) was 0.1 ng/mL, 2 ng/mL, and 0.5 ng/mL for REG, M-2, and M-5, respectively.

### 2.7. Pharmacokinetic Evaluation

Plasma data were subjected to noncompartmental pharmacokinetic analysis (NCA) using the PKanalix 2024R1 software (Lixoft, France). The maximum plasma concentration (C_max_) and time to reach C_max_ (T_max_) were obtained directly from the concentration–time curves. The elimination rate constant (k_el_) was calculated by linear regression of the final linear part of plasma concentration against time. The elimination half-life (t_0.5_) was calculated from the formula t_0.5_ = 0.693/k_el_. The area under the concentration–time curve (AUC) was calculated using the linear trapezoidal rule. All data were presented as mean ± SD.

### 2.8. Statistical Analysis

The Shapiro–Wilk test was used to determine the normality. The differences between the normally distributed variables were determined with the Student’s *t*-test. The variables that were not normally distributed were analyzed with the Mann–Whitney test. Differences were considered statistically significant when *p*-values were <0.05. Statistical analysis was conducted using the SAS software, version 9.4 (SAS Institute Inc., Cary, NC, USA).

## 3. Results

### 3.1. The Effect of PA on the Pharmacokinetics of REG, M-2, and M-5

**Figure 2:**
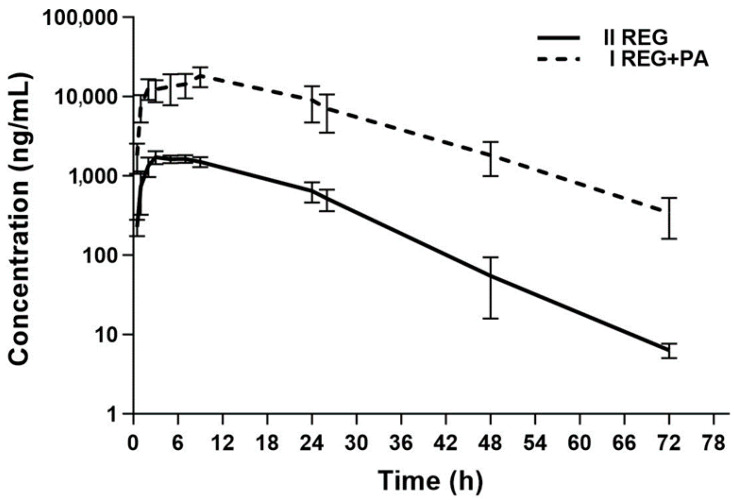
The semilogarithmic representation of the REG plasma concentration–time profiles in rats after oral administration of a single dose of *vehiculum* + REG (20 mg/kg b.w.) to the II_REG_ group [14] and REG + PA (20 mg/kg b.w. + 100 mg/kg b.w.) to the I_REG+PA_ group.

**Figure 3:**
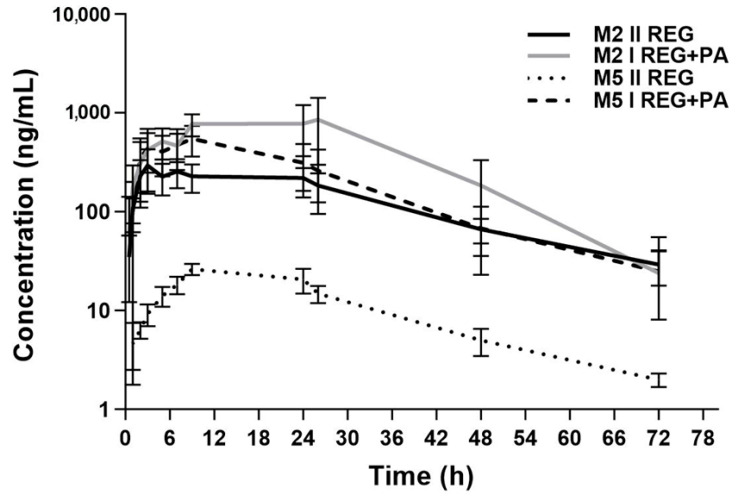
The semilogarithmic representation of the M-2 and M-5 plasma concentration–time profiles in rats receiving *vehiculum* + REG (20 mg/kg b.w.) (II_REG_) [14] and REG + PA (20 mg/kg b.w. + 100 mg/kg b.w.) (I_REG+PA_).

The plasma concentration–time curves of REG, M-2, and M-5 in the presence or absence of paracetamol are shown in Figure 2 and Figure 3. The administration of paracetamol significantly changed the pharmacokinetic profile of REG, M-2, and M-5, as summarized in Table 1. After the administration of PA, the main pharmacokinetic parameters (AUC_0–t_, AUC_0–∞_, and C_max_) of REG increased by approximately 12.31-fold, 12.44-fold, and 9.87-fold, respectively (*p* < 0.01). After the administration of PA, the C_max_, AUC_0–t_, and AUC_0–∞_ of M-2 were respectively, 3.12-, 3.23-, and 2.74-fold greater, whereas the values of the same parameters for M-5 were respectively, 22.34-, 18.74-, and 18.29-fold greater. The M-2/REG ratios for the C_max_, AUC_0–t_, and AUC_0–∞_ decreased by 6.29-fold, 3.86-fold, and 4.57-fold, respectively. The M-5/REG ratios were similar in both groups. The sum of the C_max_ values for REG, M-2, and M-5 in the I_REG+PA_ group (20,053.91 ± 6344.31 ng/mL; CV = 31.6%) was significantly greater (*p* = 0.0002; G_mean_ ratio = 10.16, and 90% CI = 6.79; CV = 15.21%) than in the II_REG_ group (2078.95 ± 691.36 ng/mL; CV = 33.3%).

### 3.2. The Effect of REG on the Pharmacokinetics of PA, GPA, and SPA

**Figure 4:**
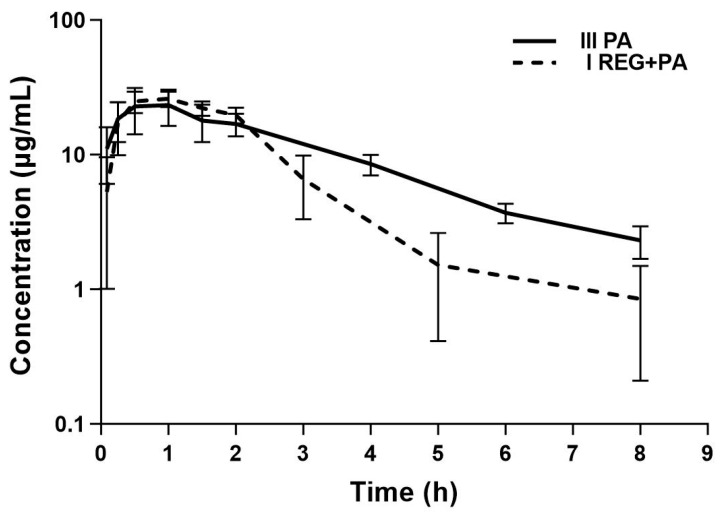
The semilogarithmic representation of the PA plasma concentration–time profiles in rats after oral administration of a single dose of *vehiculum* + PA (100 mg/kg b.w.) to the III_PA_ group [16] and REG + PA (20 mg/kg b.w. + 100 mg/kg b.w.) to the I_REG+PA_ group.

**Figure 5:**
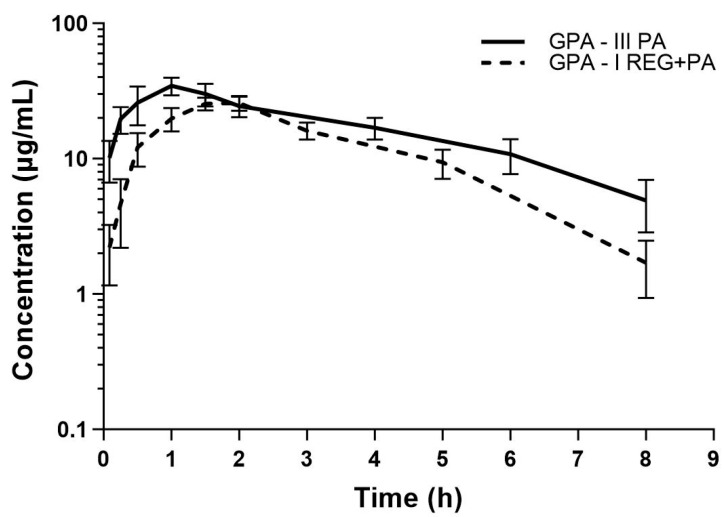
The semilogarithmic representation of the GPA plasma concentration–time profiles in rats after oral administration of a single dose of *vehiculum* + PA (100 mg/kg b.w.) to the III_PA_ group [16] and REG + PA (20 mg/kg b.w. + 100 mg/kg b.w.) to the I_REG+PA_ group.

**Figure 6:**
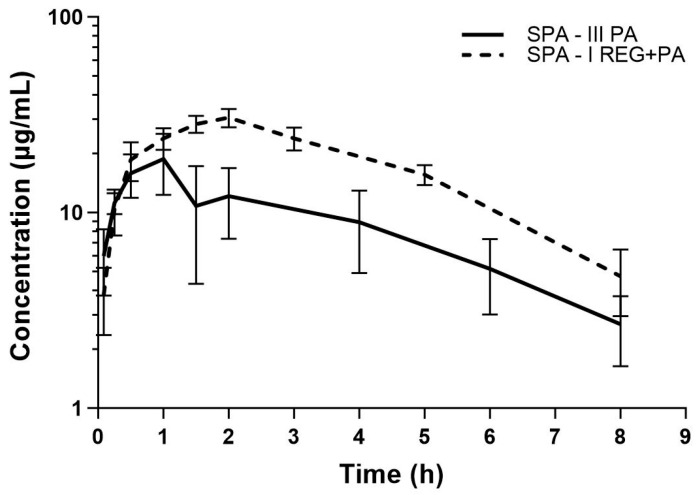
The semilogarithmic representation of the SPA plasma concentration–time profiles in rats after oral administration of a single dose of *vehiculum* + PA (100 mg/kg b.w.) to the III_PA_ group [16] and REG + PA (20 mg/kg b.w. + 100 mg/kg b.w.) to the I_REG+PA_ group.

The mean plasma concentration–time curves and the main pharmacokinetic parameters of PA, GPA, and SPA after the oral administration of PA or co-administration of REG and PA are shown in Figure 4, Figure 5 and Figure 6 and Table 2, respectively. The results showed that the AUC_0–t_ and AUC_0–∞_ for PA decreased by 20.72% when PA was co-administered with REG compared to PA alone. Statistically significant differences were revealed for V_d_/F (*p* = 0.0014), k_el_ (*p* = 0.0019), and t_0.5_ (*p* = 0.0019). The exposure to GPA was significantly lower in the presence of REG, as evidenced by the lower values of C_max_, AUC_0–t_, and AUC_0–∞_, which decreased by 41.57%, 39.16%, and 51.07%, respectively. When PA and REG were co-administered, C_max_, AUC_0–t_, and AUC_0–∞_ for SPA increased by 57.70%, 95.89%, and 91.35%, respectively. The GPA/PA ratios for AUC_0–t_ and AUC_0–∞_ were similar in both groups, but C_max_ was decreased by 63.73% in the presence of ATO. The SPA/PA ratios for AUC_0–t_ and AUC_0–∞_ increased in the I_REG+PA_ group by 139.52% and 155.32%, respectively.

## 4. Discussion

Drug interactions offer the potential to optimize cancer treatment strategies, enhancing the efficacy and bioavailability of therapeutic agents while improving their tolerability. These interactions can be crucial in refining therapy for various conditions, including cancer, by influencing the pharmacokinetic profile of drugs.

The ratio of the AUC with and without the interacting agent (perpetrator) provides a valuable tool for assessing the clinical impact of the pharmacokinetic interactions between an anticancer drug and other compounds, such as drugs or dietary components.

Specifically, an increase in AUC by fivefold or more suggests a strong inhibitory effect, while smaller increases (2–5-fold or 1.25–2-fold) indicate moderate or weak inhibition, respectively [19,20]. However, the clinical significance of many drug combinations remains unclear due to the limited availability of preclinical and clinical studies. Additionally, factors such as patient-specific characteristics—including age, renal and hepatic function, metabolic conditions, and acute illnesses—may further influence these interactions [19,20,21].

In this study, we examined the reciprocal pharmacokinetic interactions between paracetamol and regorafenib along with their active metabolites in healthy rats.

### 4.1. The Effect of PA on the Pharmacokinetics of REG, M-2, and M-5

We found that paracetamol significantly increased the bioavailability of regorafenib and its two metabolites, M-2 and M-5, in rats compared to regorafenib alone (Table 1).

The observed increase in the C_max,_ AUC_0–t_, and AUC_0–∞_ values of regorafenib (2.7-, 3.2-, and 3.2-fold, respectively) in the presence of perpetrator paracetamol may be the result of PA’s inhibition of P-gp transporter in the small intestine. The obtained values of AUC_in the presence of perpetrator_/AUC_in the absence of perpetrator_ ratios may indicate that paracetamol was a moderate inhibitor in this study. Combination therapy, consisting of a minimum of two drugs, may offer the chance of a more effective anti-cancer drug. Guo et al. [22] showed that the use of linsitinib and acetylsalicylic acid (ASA) as IGF1-R antagonists inhibited the resistance of colon cancer to regorafenib, the growth of colon-cancer-like stem cells, decreased the expression of *CD133*, *CD44*, and *CD24* genes, and increased the expression of *CDX2* and *PTEN* genes. In C57BL/6J tumor-induced mice, treatment with linsitinib, aspirin, and regorafenib increased body weight and survival and reduced blood in the feces, the number of tumors in the colon and large intestine, and the levels of inflammatory cytokines in serum and colon tissues. The study authors conclude that combination therapy with linsitinib, ASA, and REG may prevent tumor resistance or stem cell production. A favorable drug–drug interaction (regorafenib + dual JAK/HDAC small-molecule inhibitor (JAK/HDACi)) was observed in the study by Bajpai et al. [23]. They showed that the combination therapy reduced cell viability and induced cell cycle arrest in the G0–G1 phase and apoptosis of colorectal cancer cells. In addition, it synergistically reduced tumor growth and metastasis and enhanced the anti-tumor immune response. Pharmacokinetic studies have shown that the combination of these drugs increases the bioavailability of regorafenib, indicating that treatment efficacy may be due to prolonged bioavailability. A study by Zhu et al. [24] showed that administering the hepatoprotective drug Wuzhi capsule (WZC, a herbal preparation derived from *Schisandra sphenanthera*) to rats along with regorafenib resulted in a prolonged mean residence time (MRT) of the parent drug but no statistically significant difference in other pharmacokinetic parameters. While for the main metabolites of regorafenib, WZC decreased the exposure, delayed the t_max_, and prolonged the MRT of M-2 and M-5. The study authors conclude that clinicians should pay special attention to the combination of regorafenib and WZC. In addition, the levels of regorafenib and its major metabolites should be monitored during combination therapy. Recent studies show that paracetamol can reduce the effectiveness of PD-L1 inhibitors, like nivolumab, ipilimumab, pembrolizumab, or atezolizumab. High exposure to paracetamol during concomitant treatment with immune checkpoint inhibitors (ICIs) led to treatment failure and shorter survival in non-small-cell lung cancer patients. Paracetamol acted as an inhibitor of the anti-cancer immune response, and its immunomodulatory effect reduced the effectiveness of cancer therapy. The adverse effects appear to depend on the cumulative dose and duration of exposure to paracetamol. The results suggest that only substantial and/or prolonged use of paracetamol may impair the immune response to anti-PD-1/PD-L1 agents in patients with advanced NSCLC. The authors recommend that paracetamol be included in ICI therapy in no more than four doses of 1000 mg per week at the start of treatment and for the following three months [25].

### 4.2. The Effect of REG on the Pharmacokinetics of PA, GPA, and SPA

Rats receiving regorafenib and paracetamol showed an increase of approximately 53.52% in k_el_ values and a decrease in AUC_0–∞_, AUC_0–t_, t_0.5_, and V_d_/F of 23.09%, 17.16%, 49.56%, and 54.04% for paracetamol, respectively, compared to the control group. For GPA, a significantly lower level of exposure was found in the study group when compared to the control group (AUC_0–t_ = 97.9 ± 16.13 vs. 136.24 ± 23.58 µg × h/mL; AUC_0–∞_ = 102.00 ± 18.50 vs. 154.09 ± 35.58 µg × h/mL). The opposite situation was demonstrated for the metabolite SPA. A statistically significant increase in exposure was observed in group III_REG+PA_ (AUC_0–t_ = 71.92 ± 31.03 vs. 140.89 ± 17.87 µg × h/mL; AUC_0–∞_ = 82.42 ± 32.71 vs. 157.71 ± 24.35 µg × h/mL). The quantitative change in paracetamol metabolite ratios may be due to a metabolic shift toward NAPQI, but we did not determine this metabolite in our study, which is an important limitation of the work. Similar changes in the PK of paracetamol have also been observed with another protein kinase inhibitor, lorlatinib. Patients taking lorlatinib [26] showed reduced exposure to paracetamol (a drug that measures UGT) and fexofenadine (a drug that measures P-gp) compared to controls. There were decreases in AUC_0–∞_ and C_max_ of 67% and 63%, respectively, for fexofenadine, whereas there were decreases of 43% and 15%, respectively, for paracetamol. The analysis of PK changes in paracetamol may be of great clinical relevance, as its effects on cancer therapy have long been studied, including in a pharmacodynamic context. For example, a study on the risk of epithelial ovarian cancer (EOC) and paracetamol use in a Danish population of women showed a dose-dependent effect of paracetamol in reducing the risk of EOC. However, further studies are needed to confirm this [27]. This was also observed by Altinoz in his study and linked to the phenomenon of ferroptosis and the inhibition of ovarian cancer via blocking NF-κB activity by paracetamol [28]. In contrast, Weng et al. [29] presented the results of a study suggesting that paracetamol can suppress the expression of high-risk genes associated with ibrutinib resistance in patients with diffuse large B-cell lymphoma. In addition, TKIs may be used as hepatoprotective drugs during the onset of paracetamol-induced hepatotoxicity; e.g., nilotinib may have hepatoprotective effects against paracetamol toxicity due to HIF-1α inhibition [30]. The above examples of combinations of anticancer drugs with paracetamol confirm a certain potential of this drug in oncology and not only as an analgesic.

Our study has several limitations. The potential pharmacokinetic interaction between paracetamol and regorafenib after multiple doses was not investigated in this project. It is also necessary to perform studies in an induced cancer model and to determine the free fraction of regorafenib responsible for the pharmacological activity.

## 5. Conclusions

The administration of a single dose of paracetamol significantly increases the exposure to regorafenib and its active metabolites, which may exacerbate the adverse effects of the anticancer drug but may also allow the dose of regorafenib to be reduced. In contrast, regorafenib reduced the exposure to paracetamol, which may reduce the analgesic and antipyretic effects of the analgesic. Understanding the drug interactions of new anticancer drugs is important for treatment safety. In addition, this study may provide guidance for concomitant use in patients in ongoing clinical trials and in clinical practice.

## Figures and Tables

**Table 1:** Plasma pharmacokinetic parameters of REG and its metabolites M-2 and M-5 after oral administration of a single dose of *vehiculum* + REG (20 mg/kg b.w.) to the II_REG_ group [14] and REG + PA (20 mg/kg b.w. + 100 mg/kg b.w.) to the I_REG+PA_ group.

Pharmacokinetic Parameters	II_REG_ (*n* = 8) [14]	I_REG+PA_ (*n* = 8)	I_REG+PA_ vs. II_REG_ *p*-Value	G_mean_ *** Ratio (90% CI) I_REG+PA _ vs. II_REG_
REG				
C_max_ (µg/mL)	1.87 ± 0.31 (16.5)	18.45 ± 6.04 (32.7)	0.0008 *	9.51 (7.51; 12.05)
AUC_0–t_ (µg × h/mL)	36.760 ± 5.84 (15.9)	452.75 ± 166.22 (36.7)	0.0002 **	11.79 (9.35; 14.87)
AUC_0–∞_ (µg × h/mL)	36.85 ± 5.83 (15.8)	458.42 ± 167.27 (36.5)	0.0002 **	11.92 (9.46; 15.01)
t_max_ (h)	5.75 ± 2.12 (36.9)	8.25 ± 0.71 (8.6)	0.0002 **	1.70 (1.27; 2.28)
k_el_ (1/h)	0.11 ± 0.03 (22.8)	0.07 ± 0.01 (18.9)	0.0021 *	0.60 (0.49; 0.73)
t_0.5_ (h)	6.37 ± 1.33 (20.8)	10.67 ± 2.48 (23.28)	0.0005 *	1.66 (1.38; 2.01)
Cl/F (L/h × kg)	0.22 ± 0.03 (13.5)	0.02 ± 0.006 (33.52)	0.0002 **	0.08 (0.06; 0.10)
V_d_/F (L)	2.05 ± 0.61 (29.7)	0.29 ± 0.12 (40.7)	0.0002 **	0.14 (0.09; 0.19)
M-2				
C_max_ (ng/mL)	342.10 ± 132.87 (38.8)	1087.58 ± 471.76 (43.4)	0.0006 **	3.09 (2.17; 4.39)
AUC_0–t_ (ng × h/mL)	9615.36 ± 2275.41 (23.7)	31,036.39 ± 16,132.08 (52.0)	0.0002 **	2.94 (2.04; 4.25)
AUC_0–∞_ (ng × h/mL)	11,469.93 ± 2133.05 (18.6)	31,434.01 ± 16,129.53 (51.3)	0.0002 **	2.49 (1.75; 3.48)
Ratio M-2/REG				
C_max_	0.44 ± 0.73 (165.8)	0.07 ± 0.05 (72.5)	0.0019 **	0.24 (0.12; 0.49)
AUC_0–t_	0.27 ± 0.08 (29.8)	0.07 ± 0.04 (53.9)	<0.0001 *	0.25 (0.17; 0.37)
AUC_0–∞_	0.32 ± 0.09 (27.8)	0.07 ± 0.04 (53.0)	<0.0001 *	0.21 (0.14; 0.30)
M-5				
C_max_ (ng/mL)	26.23 ± 3.98 (15.2)	585.96 ± 193.12 (33.0)	0.0003 **	21.16 (15.89; 28.16)
AUC_0–t_ (ng × h/mL)	803.19 ± 177.02 (22.0)	15,054.83 ± 7479.43 (49.7)	0.0003 **	16.59 (10.86; 25.35)
AUC_0–∞_ (ng × h/mL)	847.88 ± 178.97 (21.1)	15,506.44 ± 8001.74 (51.6)	0.0003 **	16.07(10.49; 24.61)
Ratio M-5/REG				
C_max_	0.03 ± 0.06 (170.1)	0.03 ± 0.01 (28.0)	0.0541 **	1.53 (0.78; 3.00)
AUC_0–t_	0.02 ± 0.01 (23.3)	0.03 ± 0.01 (35.1)	0.0544 *	1.34 (0.97; 1.86)
AUC_0–∞_	0.02 ± 0.01 (22.8)	0.03 ± 0.01 (35.3)	0.0806 *	1.29 (0.92; 1.79)

C_max_, maximum observed plasma concentration; AUC_0–t_, area under the plasma concentration–time curve from zero to the time of last measurable concentration; AUC_0–∞_, area under the plasma concentration–time curve from zero to infinity; t_max_, time to the first occurrence of C_max_; k_el_, elimination rate constant; t_0.5_, half-life in the elimination phase; Cl/F, apparent plasma drug clearance; V_d_/F, apparent volume of distribution based on the whole curve. Arithmetic means and standard deviations (SD) are shown with coefficients of variation CV (%) in brackets. * *t*-test; ** Mann–Whitney test; *** geometric mean (G_mean_).

**Table 2:** Plasma pharmacokinetic parameters for PA, GPA, and SPA following a single *p.o.* dose of PA + *vehiculum* (100 mg/kg b.w.) to the III_PA_ group [16] and REG + PA (20 mg/kg b.w. + 100 mg/kg b.w.) to the I_REG+PA_ group.

Pharmacokinetics Parameters	III_PA_ (*n* = 8) [16]	I_REG+PA _ (*n* = 8)	I_REG+PA_ vs. III_PA_ *p*-Value	G_mean_ Ratio (90% CI) I_REG+PA _ vs. III_PA_
PA				
C_max_ (µg/mL)	24.70 ± 8.43 (34.1)	27.08 ± 3.88 (14.3)	0.4799 *	1.13 (0.92; 1.40)
AUC_0–t_ (µg × h/mL)	80.46 ± 12.10 (15.0)	66.65 ± 11.69 (17.5)	0.0359 *	0.86 (0.72; 0.95)
AUC_0–∞_ (µg × h/mL)	88.62 ± 8.96 (10.1)	68.16 ± 12.86 (18.9)	0.0024 *	0.76 (0.67; 0.87)
t_max_ (h)	0.81 ± 0.58 (71.2)	0.78 ± 0.31 (39.9)	0.8204 **	1.09 (0.62; 1.91)
k_el_ (1/h)	0.33 ± 0.10 (30.7)	0.71 ± 0.30 (42.3)	0.0019 **	2.06 (1.48; 2.85)
t_0.5_ (h)	2.26 ± 0.70 (31.0)	1.14 ± 0.45 (39.7)	0.0019 *	0.49 (0.35; 0.68)
Cl/F (mL/h × g)	0.57 ± 0.06 (10.1)	0.59 ± 0.12 (21.3)	0.7425 *	1.01 (0.87; 1.18)
V_d_/F (mL)	1.98 ± 0.63 (31.6)	0.91 ± 0.27 (29.4)	0.0014 *	0.46 (0.35; 0.61)
GPA				
C_max_ (µg/mL)	38.38 ± 3.75 (9.8)	27.11 ± 3.05 (11.25)	<0.0001 *	0.71 (0.64; 0.77)
AUC_0–t_ (µg × h/mL)	136.24 ± 23.58 (17.3)	97.9 ± 16.13 (16.5)	0.0019 *	0.72 (0.62; 0.84)
AUC_0–∞_ (µg × h/mL)	154.09 ± 35.58 (23.09)	102.00 ± 18.50 (18.1)	0.0025 *	0.67 (0.55; 0.80)
Ratio GPA/PA				
C_max_	1.67 ± 0.41 (24.8)	1.02 ± 0.19 (18.9)	0.0013 *	0.62 (0.50; 0.77)
AUC_0–t_	1.75 ± 0.47 (27.1)	1.50 ± 0.30 (19.7)	0.2285 *	0.87 (0.70; 1.09)
AUC_0–∞_	1.78 ± 0.54 (30.6)	1.53 ± 0.34 (21.9)	0.2937 *	0.88 (0.68; 1.13)
SPA				
C_max_ (µg/mL)	19.41 ± 7.05 (36.4)	30.61 ± 3.94 (12.9)	0.0015 *	1.67 (1.29; 2.15)
AUC_0–t_ (µg × h/mL)	71.92 ± 31.03 (43.2)	140.89 ± 17.87 (12.7)	<0.0001 *	2.13 (1.58; 2.86)
AUC_0–∞_ (µg × h/mL)	82.42 ± 32.71 (39.7)	157.71 ± 24.35 (15.4)	0.0002 *	2.04 (1.54; 2.71)
Ratio SPA/PA				
C_max_	0.88 ± 0.46 (52.7)	1.14 ± 0.12 (10.1)	0.1598 *	1.47 (1.04; 2.09)
AUC_0–t_	0.91 ± 0.43 (47.3)	2.18 ± 0.50 (22.9)	<0.0001 *	2.58 (1.84; 3.60)
AUC_0–∞_	0.94 ± 0.40 (42.4)	2.40 ± 0.68 (28.4)	0.0001 *	2.69 (1.94; 3.72)

C_max_, maximum observed plasma concentration; AUC_0–t_, area under the plasma concentration–time curve from zero to the time of last measurable concentration; AUC_0–∞_, area under the plasma concentration–time curve from zero to infinity; t_max_, time to the first occurrence of C_max_; k_el_, elimination rate constant; t_0.5_, half-life in the elimination phase; Cl/F, apparent plasma drug clearance; V_d_/F, apparent volume of distribution based on the whole curve. Arithmetic means and standard deviations (SD) are shown with coefficients of variation CV (%) in brackets. * *t*-test; ** Mann–Whitney test; geometric mean (G_mean_).

## Data Availability

The data presented in this study are available on request from the corresponding author.
